# Ultra-high adsorption of cationic methylene blue on two dimensional titanate nanosheets†

**DOI:** 10.1039/c8ra10172h

**Published:** 2019-02-18

**Authors:** Huiyu Yuan, Sainan Ma, Xinyu Wang, Hui Long, Xinhua Zhao, Daoyuan Yang, Wai Hung Lo, Yuen Hong Tsang

**Affiliations:** The Hong Kong Polytechnic University Shenzhen Research Institute Shenzhen People's Republic of China yuen.tsang@polyu.edu.hk; Department of Applied Physics, The Hong Kong Polytechnic University Hong Kong People's Republic of China; School of Materials Science and Engineering, Zhengzhou University Zhengzhou People's Republic of China; Department of Applied Biology and Chemical Technology, State Key Laboratory of Chinese Medicine and Molecular Pharmacology, The Hong Kong Polytechnic University Hung Hom Kowloon Hong Kong People's Republic of China

## Abstract

In this work, we examined the performance of 2D titanate nanosheets for dye adsorption. Their adsorption capacity for methylene blue (MB) is up to 3937 mg g^−1^, which is more than 10 times higher than active carbon and occupies the highest place among all the reports.

## Abstract 2

2D titanate nanosheets show ultra-high adsorption for methylene blue.

## Graphical abstract

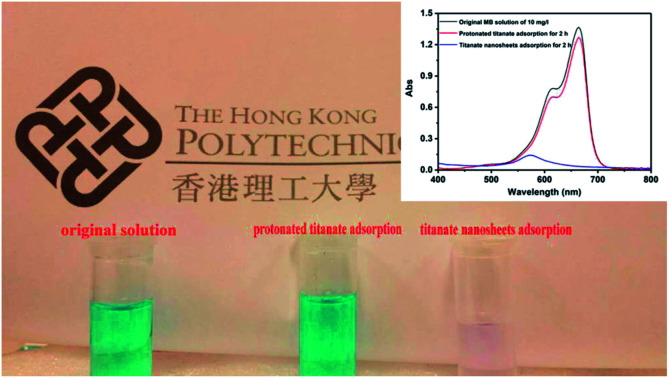

Dyes are important materials in many industries such as the textile, paper, leather, printing, and plastic industries. However, the dye effluents released by these industries give rise to major environmental issues because the dyes are generally toxic and/or carcinogenic to human beings.^1^ Dye removal from the wastewater is essential before release in these industries. There are several methods to remove dye contaminants from wastewaters, such as adsorption, coagulation, chemical oxidation, membrane separation processes, *etc.*^1^ Among the technologies, the adsorption process has been generally considered to be the most efficient method of quickly lowering the concentration of dissolved dyes in an effluent.^2^ Various adsorbents have been studied for dye removal purposes, such as activated carbons (ACs),^3–6^ clay,^7,8^ sawdust,^7^ beer brewery waste,^2^ chitosan,^9^ metal oxides,^10–12^ carbon nanotubes and graphene oxide.^13,14^ A general strategy to enhance the dye adsorption is to increase the surface area of the adsorbents, so the nanosized materials have been attracting much attention for the dye removal application because they exhibit much large surface area.^14–20^

Two dimensional (2D) materials are gaining great attention since discovery of graphene.^21–23^ The 2D materials consist of large number of groups, such as graphene, transition metal dichalcogenides (TMDs), transition metal oxides (TMOs), layered double hydroxides (LDHs), MXene *et al.*^21,24–26^ One may divide the 2D materials into three groups based on the charge of the host layer: neutral, negatively charged, and positively charged. The charged 2D materials must couple with counterions to compensate the charges. As a sample, 2D titanium oxide, also called titanate nanosheet (TONS) is negatively charged. The TONS is generally synthesized by the chemical exfoliation.^27,28^ These chemical exfoliated TONS is monolayer nanocrystals with crystallographic thickness of 0.75 nm.^29^ The nanosheets in aqueous solution behave as individual molecular entities, and they are surrounded by positively charged ions (TBA^+^ and H^+^).^28,30,31^ The colloidal system is governed by electrostatic interaction.^31^

As an emerging class of materials, the charged 2D materials are potentially useful for treatment of the dyes with opposite charges. This has motivated us to study the performance of the titanate nanosheets, a charged 2D material, for dye treatment.

We synthesized the titanate nanosheets *via* top down approach report by Sasaki *et al.*^32,33^Fig. 1a and b show the XRD pattern and SEM image of the protonated titanate. The XRD pattern is consistent with the previous report by Sasaki *et al.*^32,33^ The SEM image shows that the crystal size is up to 20 μm. Fig. 1c shows the UV-Vis spectrum of the exfoliated titanate nanosheets. The peak is located at 265 nm, which is consistent with the value in the previous reports.^28,29,34^Fig. 1d shows the STEM image of the nanosheets obtained by chemical exfoliation, and provides an evidence for the successful exfoliation. The AFM image of the nanosheets transferred to Si substrate by simple adsorption shown in Fig. S1† shows the lateral size of the adsorbed nanosheets is generally less than 1 μm.

**Fig. 1:**
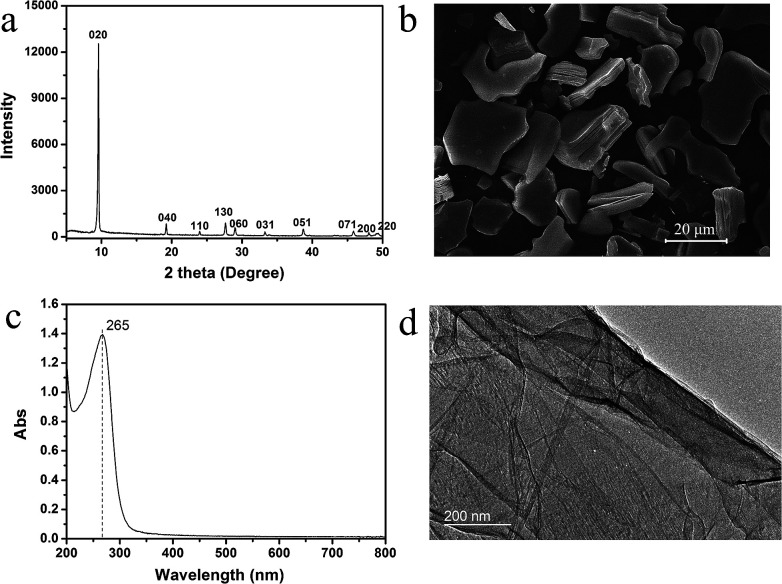
The XRD (a), SEM image (b) of protonated titanate; UV-Vis spectrum (c) and STEM image (d) of 2D titanate nanosheets. The dash line in (c) indicates the position of 265 nm.

We first evaluated the adsorption capacity of the exfoliated nanosheets comparing with its bulk counterparts K_0.8_[Ti_1.73_Li_0.27_O_4_] (KLTO) and its protonated form (HTO). Fig. 2a shows that the adsorption capacity and adsorption percentage of the TONS in the function of the initial concentration of MB. The observed adsorption capacity increased up to 2236 mg g^−1^ as the initial MB concentration was 4000 mg l^−1^. It is noted that this value is underestimated because we didn't consider the MB loss in experimental process. We analyzed the adsorption isotherm by fitting it with the Freundlich and the Langmuir models and the fitting curves are shown in Fig. 2b. The Langmuir model was fitting better than the Freundlich model, and the correlation coefficients *R*^2^ for the Langmuir and the Freundlich models are 0.97 and 0.93, respectively. The better Langmuir model fitting indicates that the adsorption of MB on TONS surface is homogenous.^11^ The maximum adsorption capacity fitted by the Langmuir model is 3937 mg g^−1^. This adsorption capacity is, to the best of our knowledge, the largest for the MB adsorption among all the reports (see Table S1 in the ESI†).^1,15–17,35^ However, the dye removal efficiency is low even at the low initial concentration. Only half even less dye can be adsorbed as shown in Fig. 2a. Fig. 2c shows the morphology of the TONS after the MB adsorption at initial concentration of 1000 mg l^−1^. The 2D feature of the TONS is clearly presented, indicating its good structural stability. In contrast to the large adsorption capacity of the TONS, its layered counterparts KLTO and HTO show limited adsorption capacity as shown Fig. 2d (see the adsorption data for KLTO in Fig. S2†). Our data show that the adsorption capacities of the KLTO and HTO are 3.45 and 2.63 mg g^−1^, respectively.

**Fig. 2:**
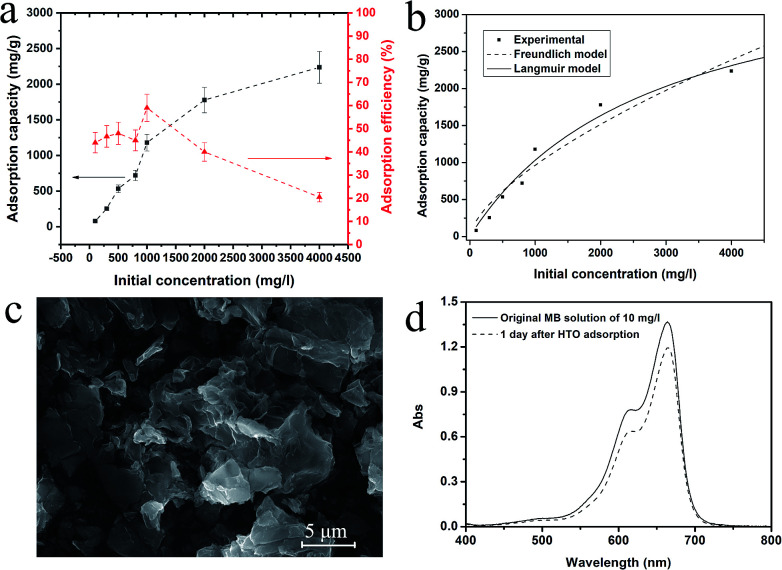
(a) The adsorption capacity and adsorption efficiency of titanate nanosheets at different initial concentrations; (b) the adsorption isotherm fitting with the Freundlich and the Langmuir models; (c) the typical morphology of TONS after MB adsorption (the initial concentration at 1000 mg l^−1^); (d) the UV-Vis spectra of MB solution with concentration of 10 mg l^−1^ before and after HTO adsorption.

Fig. 3a presents the UV-Vis spectra of the MB (10 mg l^−1^) and the TONS solution centrifuged at different time. The TONS shows rapid adsorption of the MB molecules. The concentration of the MB monomers dropped to a negligible concentration within 1 min, and a new peak appeared at ∼575 nm (purple by naked eyes) which can be assigned to the trimer form of the MB.^36–38^ It is noted that the adsorption of MB monomers onto the TONS is much faster than other material systems, such as clay and graphene oxide.^14,39^ The reason beyond this phenomenon is probably due to the large surface area and high charge density. It has been reported that the external adsorption of the MB is a fast process comparing with the internal adsorption in the clay system.^39^ In this work, the TONS were dispersed in aqueous solution, so the external adsorption was dominating in the adsorption process. Another interesting feature is that the concentration of the MB trimers increased as the time proceeded. Because the MB monomers had been adsorbed onto the TONS within 1 min, the increase of the MB trimer concentration after 1 min suggests that the MB trimers came from desorption of the MB from the TONS. We analyzed the kinetic of trimer formation by assuming that the MB^+^ ions participating formation of trimer are irrelevant to the kinetic of desorption of MB–TONS complex, and we found the third order kinetic model is the best fitting to the experimental data among first, second and third order kinetic models, see Fig. 3b. The third order kinetic model yielded the correlation coefficient value of 0.965, while the first and second order kinetic models yielded the correlation coefficient values of 0.909 and 0.948, respectively. The data suggest that recombinative molecular desorption occurred, that is, multiple MB molecules desorbed and formed trimers. The desorption of counterions on the TONS was also observed with the TBA^+^ ions because the TBA–TONS complex went through an ionization process in the course of time.^40^ The data here suggest the MB–TONS complex also went through an ionization process in the course of time after the rapid combination.

**Fig. 3:**
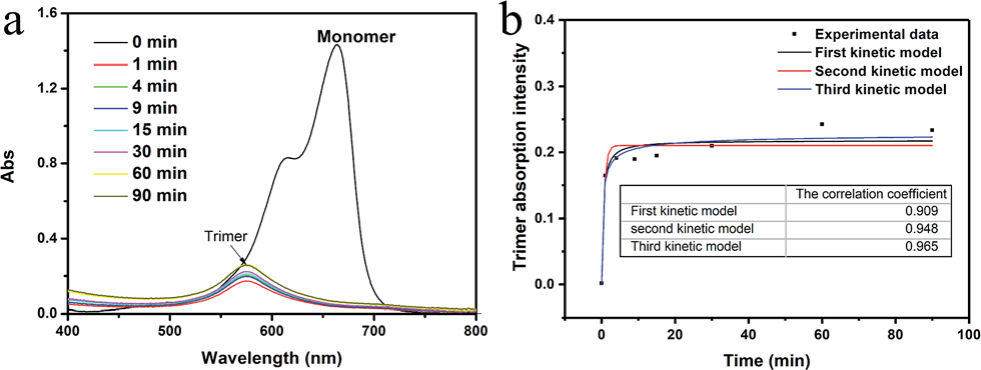
(a) UV-Vis spectra of MB and titanate nanosheets solution centrifuged at different time. (b) The trimer adsorption intensity *vs. t* for describing the desorption kinetics of methylene blue (10 mg l^−1^) on the TONS.

We also investigated the effect of the initial concentration of the MB on adsorption. Fig. 4a shows the UV-Vis spectra of residual (equilibrium) solution at different initial concentrations. The data show that the trimers were the main product after desorption at low concentrations (<100 mg l^−1^), while the dimers (peak position at ∼615 nm) appeared and became more and more pronounced at high concentrations (>300 mg l^−1^). We plotted the intensity change of monomers and dimers in reference to trimers in Fig. 4b. It is clear that the intensity of monomers didn't change significantly in reference to trimers, but the intensity of the dimers did when the initial concentration of the MB solution increased, especially at 500 mg l^−1^. It is noted that one should not compare the absolute values instead of the trend in the curves. The right vertical axis in the Fig. 4b presents the ratio of active surface area of adsorbed MB : TONS in the function of initial MB concentration in our study. We calculated the values assuming the MB molecules have only one side active to occupy the surface of the TONS. Fig. 4b shows that the number of the adsorbed MB^+^ ions was sufficient to cover the entire TONS surface and the second layer of the MB^+^ formed on the TONS surface when the initial concentration of MB solution reached 300 mg l^−1^. Taking into consideration that the dimer started forming at the initial concentration of 300 mg l^−1^, (see Fig. 4a) and the dimer formation enhanced further as the MB^+^ concentration increased, these data suggest that the formation of first layer MB^+^ on TONS surfaces suppresses the formation of trimers and promotes the formation of dimers. This is reasonable because the formation of first layer MB^+^ reduces the charge density of the MB–TONS complex. Bujdák *et al.* report that the high charge density of clay induces high order agglomeration of MB dye and low charge density suppresses the agglomeration.^41^ In our study, the charge density of the TONS remains constant, but the surface charge density of nanosheet complex changes with different amount of MB adsorption. The charge density of the MB–TONS complex determines the yield either trimer or dimer.

**Fig. 4:**
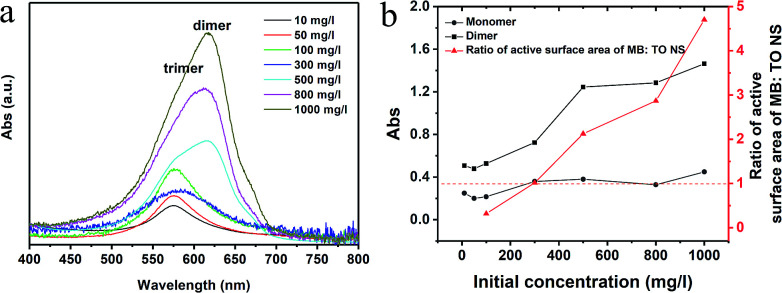
(a) UV-Vis spectra of residual (equilibrium) solution, (b) the intensity change of the monomer and dimer in the reference with trimer and the ratio of active surface area of adsorbed MB : TONS (the red dash line marks the position of left vertical axis at 1).

In these colloidal systems, the TONS complex is governed by electrostatic force.^31^ According to the double layer theory, the TONS should be tightly bounded with the counterions such as MB^+^, TBA^+^ and H^+^ in first layer. In our solutions which are very basic, the concentration of these ions is in following order, TBA^+^ (0.0129 mol l^−1^) > MB^+^ (0.00313 mol l^−1^ for 1000 mg l^−1^) ≫ H^+^. So, the TBA^+^ and MB^+^ ions are the main contributors to build the first layer. Given the size of the MB^+^ molecules 1.7 × 0.76 × 0.33 nm, the charge density is calculated to be 1.78*q*_e_/nm^2^ (where *q*_e_ is the electron charge) in the form of perpendicular to the TONS.^42^ In reality, the charge density should be smaller than this value because MB molecules are preferable to bound with TONS at certain angle. For example, the angle was found to be 65–70 °C on mica surface.^41^ The charge density of the TBA^+^ and NSTO is 1.63 and 4.72*q*_e_/nm^2^, respectively.^28^ Therefore, one layer of the TBA^+^ and MB^+^ ions is insufficient to compensate the charge on the TONS surface. The TBA^+^ and MB^+^ ions in second and outer layers are necessary, but are loosely attracted to the TONS by the Coulomb force. The large dye adsorption capacity is probably due to this multilayer dye molecule adsorption behavior and the large surface area of the TONS. The mechanism of the ultra-high adsorption is to be verified.

In summary, we studied the charged TONS for the MB adsorption treatment. We found that the TONS possess an ultra-high dye adsorption, and the adsorption capacity of titanate nanosheets is found up to 3937 mg g^−1^. However, our data show that the dye removal efficiency of titanate nanosheets is low as less than 50%. Based on our analysis, we conclude that the large dye adsorption capacity is due to the large surface area of the TONS and the multilayer dye molecule adsorption behavior because of the presence of the surface charge. Furthermore, our study shows that the adsorption is a rapid process, and the loosely adsorbed MB molecules go through desorption to form high order agglomerates in the course of time. Our analysis indicates the desorption process a recombinative molecular desorption. What's more, the product after desorption varies with the initial concentration of MB. Low concentration yields trimers, and high concentration promotes formation of dimers.

It is well known that MB is a well-used redox indicator for photocatalysis study, at the end, we would like to highlight that one should use MB as the indicator more carefully because the color change may not be always due to the catalytic effect.

## Conflicts of interest

There are no conflicts to declare.

## Supplementary Material

RA-009-C8RA10172H-s001
