# Supplementary material for: Ultra-high adsorption of cationic methylene blue on two dimensional titanate nanosheets
Source: RSC Adv. 2019 Feb 18;9(11):5891–4. doi: 10.1039/c8ra10172h (PMC9060896; doi:10.1039/c8ra10172h)
Supplement: RA-009-C8RA10172H-s001 [file RA-009-C8RA10172H-s001.pdf]

Electronic Supplementary Material (ESI) for RSC Advances.
This journal is © The Royal Society of Chemistry 2019

Supplementary Information

Ultra-high adsorption of cationic Methylene Blue on two dimensional titanate nanosheets

Huiyu Yuan[a,b,c], Sainan Ma[b], Xinyu Wang[b], Hui Long[a,b], Xinhua Zhao[b], Daoyuan Yang[c], Wai Hung Loc[d], Yuen Hong Tsang[a,b]†

[a] The Hong Kong Polytechnic University Shenzhen Research Institute, Shenzhen, People's Republic of China

[b] Department of Applied Physics, The Hong Kong Polytechnic University, Hong Kong, People's Republic of China

[c] School of Materials Science and Engineering, Zhengzhou University, Zhengzhou, People's Republic of China

[d] Department of Applied Biology and Chemical Technology and State Key Laboratory of Chinese Medicine and Molecular Pharmacology, The Hong Kong Polytechnic University, Hung Hom, Kowloon, Hong Kong, People's Republic of China

†E-mail: yuen.tsang@polyu.edu.hk

1. MATERIALS AND METHODS

1.1 Materials

Titanium(IV) dioxide TiO_2, anhydrous potassium carbonate K_2CO_3, and lithium carbonate Li_2CO_3 had a purity of 97.0% or higher and were used as received. Tetra n-butylammonium hydroxide TBAOH (40 wt % H_2O, Alfa Aesar) were used as received. Methylene blue had a purity of 98.5 and was used as received. Demineralized water was used throughout the experiments.

1.2 Synthesis of titanate nanosheets

Titanate nanosheets were synthesized by chemical exfoliation of layered protonated titanate (HTO). In detail, the layered precursor $K_{0.8}[Ti_{1.73}Li_{0.27}O_4]$ (KLTO) was obtained by sintering of K_2CO_3, $LiCO_3$ and TiO_2 at 1000 °C for 20 h according to Sasaki's method.[32] Then, 3 g of the KLTO powders was protonated in 2 mol/l HNO_3 solution (300 ml) at room temperature while stirring. After treatment for 3 days, the acid exchanged crystals of KLTO were collected by filtration and washed with a copious quantity of pure water, and then air-dried to get HTO powders. The exfoliation of HTO crystals was carried out by reaction with a TBAOH solution. The HTO powders (0.1 g) were mixed with TBAOH and water to a TBAOH/H^+ (H^+ refers to the protons of HTO powder) ratio of 4/1 in 10 mL solution. The titanate nanosheets (TONS) solutions were stirred for 1 day, and then are ready for the adsorption experiments.

1.3 Methylene blue adsorption

The details of the kinetic experiment were given below and all the experiments were carried out under 298 K at ambient conditions. The MB aqueous solution with concentration of 10 mg/L was prepared prior to the experiment. 100 ml of the MB aqueous solution was used and vigorously stirred in a beaker. Then 5 ml TONS solution was added to the beaker. 4 ml of the mixtures at mixing time of 1, 4, 9, 15, 30, 60 and 90 min was centrifuged at 12000 rpm and the suspension was collected for further analysis.

For the isotherm adsorption experiments, the MB aqueous solutions with concentration of 50, 100, 300, 500, 800, 1000, 2000, and 4000 mg/l were prepared, respectively, prior to the experiment. 100 ml of the MB aqueous solutions were used and vigorously stirred in a beaker. Then 5 ml TONS solution was added to the beaker and was allowed to mix for 40 h. While the mixing, the beaker was sealed to prevent water evaporation. After 40 h mixing, the mixture solution was centrifuged at 12000 rpm, and then the suspension were collected for further analysis. And the residues were dried at 60°C for 2 days for further analysis. The adsorption capacity of TONS in this study was calculated by following equation,

$$qe = (m_{residue}-m_{TONS})/ m_{TONS}$$

Where, qe is the amount of MB dye adsorbed at equilibrium time (mg/g), $m_{residue}$ is the weight of the residue and m_{TONS} is the effective weight of the TONS. Because of the materials loss in the process, the effective weight of the TONS is less than the materials added. Our statistic data show that 20% materials loss applied.

The adsorption capacities of the KLTO and HTO were calculated by following equation,

$$qe,bulk = (C_0-Ceq)*V/m$$

Where qe,bulk is the adsorption capacities of the KLTO or HTO at equilibrium time, C_0 represents the initial MB concentration, Ceq is the equilibrium MB concentration in solution after adsorption, V is the volume of the aqueous solution and m is the mass of the KLTO or HTO.

The adsorption efficiency of the TONS was calculated by following equation,

$\eta = (m_{residue}\text{-}m_{TONS})/m_{MB}$

Where, η is the adsorption efficiency at equilibrium time, $m_{residue}$ is the weight of the residue, m_{TONS} is the effective weight of the TONS, and m_{MB} is the mass of MB in the initial solution.

1.4 Characterization

Powder X-ray diffraction (XRD) data were acquired on a Rigaku smartlab (Cu Kα radiation with a wavelength of 0.15405 nm). Scanning electron microscopy (SEM; Tescan VEGA3) was used to acquire information on the particle size and morphology. Scanning transmission electron microscopy (STEM; Joel JEM-2100F) was used to visualize the nanosheets. Atomic force microscopy (AFM; Bruker NanoScope 8) was used to visualize the nanosheets deposited on Si substrates. The AFM data were further analyzed using the Gwyddion software package. UV−vis spectra of samples were recorded with a Cary 50 UV−vis spectrophotometer in transmission mode. All the solutions were diluted to an appropriate concentration for UV-vis spectroscopic measurements except for the 10 mg/l solutions.

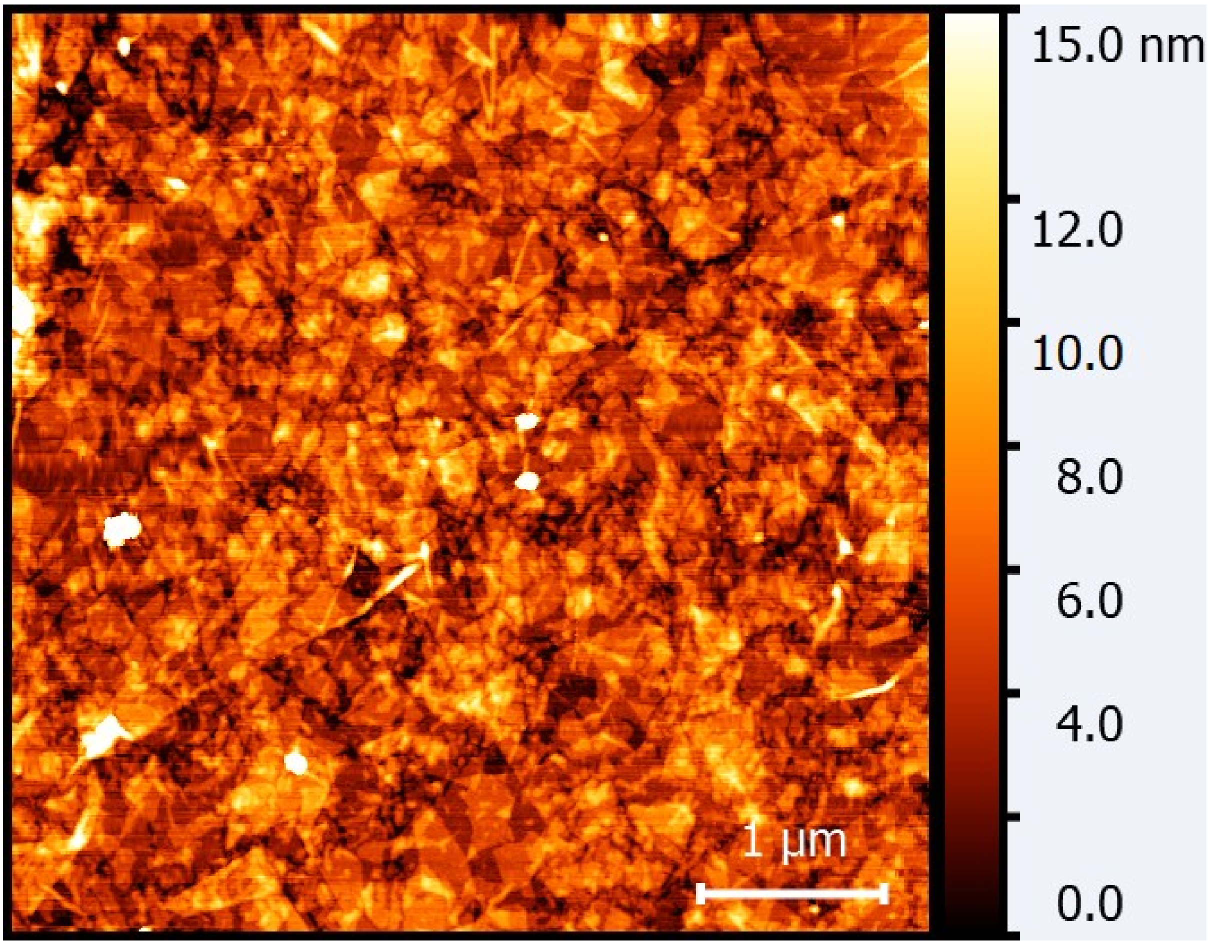

Figure S1 the AFM image of 2D titanate nanosheets.

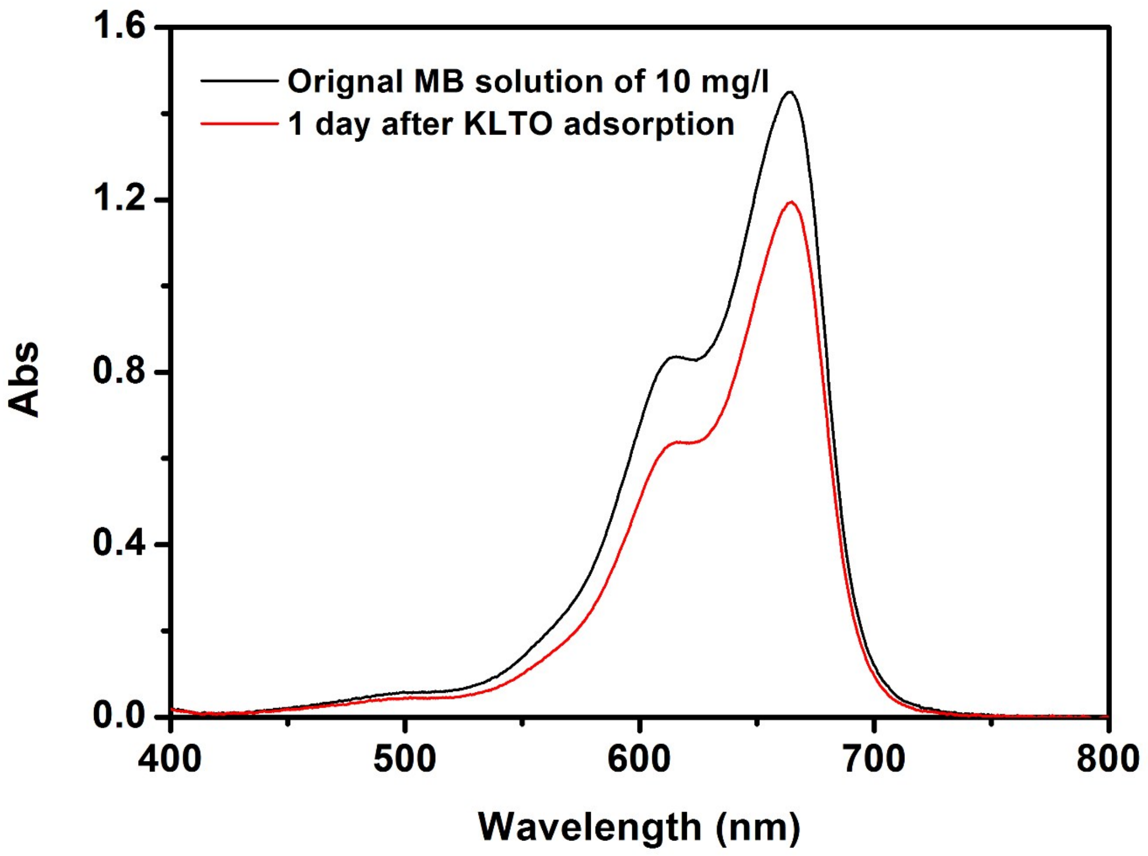

Figure S2 the UV-Vis spectra of MB solution with concentration of 10 mg/l before and after KLTO adsorption.

Table A. 1 Comparison of the Methylene Blue adsorption capacity from literature and this work

Adsorbent	Specific surface area (m^2/g)	The maximum adsorption capacity	Ref.
Active carbon	1688	270.27 (293 K)	1[1]
Graphene oxide	32	243.90 (293 K)	
Carbon nanotube	177	188.68 (293 K)	
Oil palm fibre activated carbon	1354	277.78 (305 K)	2[2]
Montmorillonite clay	62	289.12 (310 K)	3[3]
Graphite oxide	28.5	350 (300 K)	4[4]
Activated carbon produced from flamboyant pods	2854	889.58	5[5]
Fe_3O_4@C nanoparticles	--	44.38	6[6]
Humic acid-coated Fe_3O_4 nanoparticles	--	93	7[7]
Montmorillonite clay	118.1	71.12	8[8]

modified with iron oxide			
Graphene oxide-sodium alginate gel	18.98	833.3 (303 K)	9[9]
Surface hydroxyl group enriched TiO_2 nanotube	196.5	57.14 (303 K)	10[10]
Graphene-carbon nanotube composite	--	65.79 (283 K)	11[11]
Rhamnolipid-functionalized graphene oxide	42.46	529.10 (298 K)	12[12]
Graphene oxide	--	598.8 (305 K)	13[13]
Fe_3O_4 /activated montmorillonite nanocomposite	147.92	106.38 (293 K)	14[14]
Titanate nanosheets	610.5 (theoretical value)	3937 (303 K)	This study

References

[1] Y. Li, Q. Du, T. Liu, X. Peng, J. Wang, J. Sun, Y. Wang, S. Wu, Z. Wang, Y. Xia, Comparative study of methylene blue dye adsorption onto activated carbon, graphene oxide, and carbon nanotubes, Chemical Engineering Research and Design 91 (2013) 361-368.

[2] I. Tan, B. Hameed, A. Ahmad, Equilibrium and kinetic studies on basic dye adsorption by oil palm fibre activated carbon, Chemical Engineering Journal 127 (2007) 111-119.

[3] C.A.P. Almeida, N.A. Debacher, A.J. Downs, L. Cottet, C.A.D. Mello, Removal of methylene blue from colored effluents by adsorption on montmorillonite clay, Journal of Colloid and Interface Science 332 (2009) 46-53.

[4] P. Bradder, S.K. Ling, S. Wang, S. Liu, Dye adsorption on layered graphite oxide, Journal of Chemical & Engineering Data 56 (2010) 138-141.

[5] A.M.M. Vargas, A.L. Cazetta, M.H. Kunita, T.L. Silva, V.C. Almeida, Adsorption of methylene blue on activated carbon produced from flamboyant pods (Delonix regia): Study of adsorption isotherms and kinetic models, Chemical Engineering Journal 168 (2011) 722-730.

[6] Z. Zhang, J. Kong, Novel magnetic Fe_3O_4@C nanoparticles as adsorbents for removal of organic dyes from aqueous solution, Journal of Hazardous Materials 193 (2011) 325-329.

[7] X. Zhang, P. Zhang, Z. Wu, L. Zhang, G. Zeng, C. Zhou, Adsorption of methylene blue onto humic acid-coated Fe3O4 nanoparticles, Colloids and Surfaces A: Physicochemical and Engineering Aspects 435 (2013) 85-90.

[8] L. Cottet, C.A.P. Almeida, N. Naidek, M.F. Viante, M.C. Lopes, N.A. Debacher, Adsorption characteristics of montmorillonite clay modified with iron oxide with respect to methylene blue in aqueous media, Applied Clay Science 95 (2014) 25-31.

[9] T. Ma, P.R. Chang, P. Zheng, F. Zhao, X. Ma, Fabrication of ultra-light graphene-based gels and their adsorption of methylene blue, Chemical Engineering Journal 240 (2014) 595-600.

[10] T.S. Natarajan, H.C. Bajaj, R.J. Tayade, Preferential adsorption behavior of methylene blue dye onto surface hydroxyl group enriched TiO2 nanotube and its photocatalytic regeneration, Journal of Colloid and Interface

Science 433 (2014) 104-114.
[11] P. Wang, M. Cao, C. Wang, Y. Ao, J. Hou, J. Qian, Kinetics and thermodynamics of adsorption of methylene blue by a magnetic graphene-carbon nanotube composite, Applied Surface Science 290 (2014) 116-124.
[12] Z. Wu, H. Zhong, X. Yuan, H. Wang, L. Wang, X. Chen, G. Zeng, Y. Wu, Adsorptive removal of methylene blue by rhamnolipid-functionalized graphene oxide from wastewater, Water Research 67 (2014) 330-344.
[13] H. Yan, X. Tao, Z. Yang, K. Li, H. Yang, A. Li, R. Cheng, Effects of the oxidation degree of graphene oxide on the adsorption of methylene blue, Journal of Hazardous Materials 268 (2014) 191-198.
[14] J. Chang, J. Ma, Q. Ma, D. Zhang, N. Qiao, M. Hu, H. Ma, Adsorption of methylene blue onto Fe_3O_4/activated montmorillonite nanocomposite, Applied Clay Science 119 (2016) 132-140.
